# Physicochemical and Mechanical Properties of Bis-GMA/TEGDMA Dental Composite Resins Enriched with Quaternary Ammonium Polyethylenimine Nanoparticles

**DOI:** 10.3390/ma14082037

**Published:** 2021-04-18

**Authors:** Izabela M. Barszczewska-Rybarek, Marta W. Chrószcz, Grzegorz Chladek

**Affiliations:** 1 Department of Physical Chemistry and Technology of Polymers, Faculty of Chemistry, Silesian University of Technology, 44-100 Gliwice, Poland; Marta.Chroszcz@polsl.pl; 2 Department of Engineering Materials and Biomaterials, Faculty of Mechanical Engineering, Silesian University of Technology, 44-100 Gliwice, Poland; Grzegorz.Chladek@polsl.pl

**Keywords:** quaternary ammonium polyethylenimine derivatives, nanoparticles, dental composite, mechanical properties

## Abstract

Modification of dental monomer compositions with antimicrobial agents must not cause deterioration of the structure, physicochemical, or mechanical properties of the resulting polymers. In this study, 0.5, 1, and 2 wt.% quaternary ammonium polyethylenimine nanoparticles (QA-PEI-NPs) were obtained and admixed with a Bis-GMA/TEGDMA (60:40) composition. Formulations were then photocured and tested for their degree of conversion (DC), polymerization shrinkage (S), glass transition temperature (T_g_), water sorption (WS), solubility (SL), water contact angle (WCA), flexural modulus (E), flexural strength (σ), hardness (HB), and impact resistance (a_n_). We found that the DC, S, Tg, WS, E, and HB were not negatively affected by the addition of QA-PEI-NPs. Changes in these values rarely reached statistical significance. On the other hand, the SL increased upon increasing the QA-PEI-NPs concentration, whereas σ and a_n_ decreased. These results were usually statistically significant. The WCA values increased slightly, but they remained within the range corresponding to hydrophilic surfaces. To conclude, the addition of 1 wt.% QA-PEI-NPs is suitable for applications in dental materials, as it ensures sufficient physicochemical and mechanical properties.

## 1. Introduction

The development of dental materials with antibacterial activity represents a challenge in modern biomaterial science engineering [1,2]. Currently used commercial dental restorative composites ensure satisfactory mechanical properties, biocompatibility, aesthetics, and economics [3,4]. One of the biggest remaining problems is the marginal gap formation between the restoration and the adjacent tooth tissue due to polymerization shrinkage [5,6,7]. The narrow gap width (clinically acceptable marginal gaps vary between 30 and 200 µm [8]) creates a favorable place for dental plaque accumulation [7,9]. Bacterial metabolic processes lead to the emergence of secondary caries, inflammation reactions, and in extreme cases, dental restoration failure [9,10,11].

The most frequent method for providing dental restorative composites with anti-bacterial properties is a physical modification by admixing a bioactive compound [1]. Several low molecular weight inorganic and organic substances have been successfully added to commercial dental restorative composites to achieve materials with antimicrobial activity. Zinc oxide [9,12,13], titanium dioxide [14,15], calcium phosphate [16,17], gold, and silver [18,19] are the most popular examples of the first group. The latter group is represented by antibiotics [20], chlorhexidine [9,21,22], furanone, ursolic acid, benzalkonium chloride, triclosan, and methacryloyloxydodecyl pyridinium bromide [9]; however, toxic responses of tissues adjacent to the reconstructions modified with these substances are often reported [12,13,14,15,16,17,18,19,20,21,22].

A need for less cytotoxic antibacterial agents has led to the development of dental materials based on quaternary ammonium compounds (QACs). The first proposals of QACs usage in dentistry included bioactive compositions for external applications onto the surfaces of teeth, such as the protective tooth coating consisting of polyethylenimine (PEI) quaternized with fatty acid [23], and cleaning composition comprising PEI and low molecular weight quaternary ammonium biocide (alkyltrimethylammonium halide) [24]. The short-lasting antibacterial effect of these materials has disqualified them from common usage. The long-lasting antibacterial effect can be achieved by the chemical modification of dental dimethacrylate matrices with mono- and dimethacrylate monomers having the quaternary ammonium group. They are covalently bonded to a composite matrix due to copolymerization with dental dimethacrylates. This solution represents a dynamically developing research area; however, materials of satisfactory physicomechanical performance have not been achieved so far [25]. Other studies are conducted on the physical modification of dental composites by admixing particles functionalized with quaternary ammonium groups. The initial idea comprised the application of a bioactive filler, achieved by grinding of a methacrylate-silica composite whose matrix was modified by copolymerization with methacryloyloxydodecylpyridinium bromide (MDPB). The antibacterial activity of that material was weak due to the screening of quaternary ammonium groups [26]. To overcome the disadvantages of the MDPB-based filler, crosslinked quaternary ammonium polyethylenimine nanoparticles (QA-PEI-NPs) were synthesized [27]. Their polycationic character resulted in a higher antibacterial activity compared with the MDPB-based filler, allowing QA-PEI-NPs to be used in smaller amounts [1].

**Figure 1:**
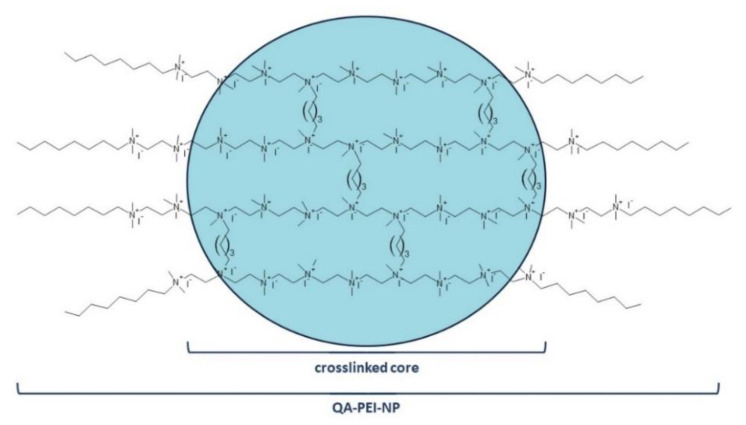
The chemical structure of QA-PEI-NPs.

QA-PEI-NPs are obtained from linear polyethylenimine (PEI) in a three-stage process that includes crosslinking, telomerization, and quaternization. In the first stage, linear PEI is crosslinked with an alkyl dihalide, usually 1 to 20 mol.% 1,5-dibromopentane. This reaction results in the formation of the insoluble nanoparticle core from which short PEI chains protrude. They are terminated with primary amino end groups that are subjected to telomerization. The two methods that may be used alternatively include N-alkylation and reductive amination, but N-alkylation is primarily used when octyl bromide is used as the N-alkylation agent. This step determines the hydrophobicity of the nanoparticles, which is responsible for the interactions between QA-PEI-NPs and lipids constituting bacterial membranes. Quaternization, also called N-methylation, is performed in the final stage. The quaternary ammonium groups are created during the reaction of secondary and tertiary amino groups with iodomethane. Their presence provides QA-PEI-NPs with antibacterial activity (Figure 1) [1,27,28,29].

The antimicrobial activity of QA-PEI-NPs has been widely examined [1]. It has been demonstrated in many studies that QA-PEI-NPs exert antibacterial activity against many bacteria strains. The Gram-positive bacteria, such as *Staphylococcus aureus* [29,30,31] and *Streptococcus mutans* [32] showed greater susceptibility to the antibacterial activity of QA-PEI-NPs than the Gram-negative bacteria, such as *Escherichia coli* [33] and *Pseudomonas aeruginosa* [29,31]. In those studies, the influence of the crosslink density, N-alkylation agent, N-alkylation degree, and quaternization degree on the antibacterial activity of QA-PEI-NPs was also demonstrated. It can be concluded that the highest efficiency could be achieved using 1,5-dibromopentane as the crosslinker in a ratio of 0.04 mol/mol PEI [32], 1-bromooctane N-alkylation agent in the ratio of 1 mol/mol PEI [29,30,32], and iodomethane quaternization agent in the ratio of 3 mol/mol PEI [30]. 

In other studies, the antibacterial activity of commercially available dental composite restorative materials enriched with QA-PEI-NPs was investigated. Dimethacrylate resin-based composites modified with 0.5 to 2 wt.% QA-PEI-NPs showed activity against the following bacterial strains: *S. mutans*—the bacteria identified the most with tooth decay [28,32,34,35], *S. aureus* [36], *P. aeruginosa* [36], *E. coli* [36], *Staphylococcus epidermidis* [36], *Enterococcus faecalis* [35,36,37,38], *Actinomyces viscosus* [34,35], and *Lactobacillus casei* [35]. It was found that 1 wt.% QA-PEI-NPs was sufficient to achieve a good antibacterial effect against the Gram-positive bacteria *S. mutans*, *S. aureus*, *A. viscosus*, *E. faecalis*, and *L. casei*. This concentration did not alter the cytotoxicity of the original material and did not exert harmful effects on living tissues. The Gram-positive bacteria, *S. epidermis* as well as Gram-negative bacteria, *P. aeruginosa* and *E. coli*, showed weaker responses to the presence of QA-PEI-NPs, and their higher content (2 wt.%) was required to achieve complete bacterial growth inhibition. The presence of 2 wt.% QA-PEI-NPs slightly increased the material’s cytotoxicity [36,39,40].

Based on the above, QA-PEI-NPs have been shown as highly efficient antibacterial agents for dental restorative materials based on dimethacrylate resins; however, there is still not enough knowledge of the influence of how QA-PEI-NPs affect the structural, physicochemical, and mechanical properties of this type of dental material matrix. It is well known that the deterioration of any property can negatively affect the composite performance and even reduce its lifetime [41]; therefore, this expertise is required for the development of new dental restorative materials modified with QA-PEI-NPs toward solutions aimed at achieving a set of optimum characteristics.

The degree of conversion (DC) of double bonds is a particularly important structural property for the proper functioning of dental materials based on dimethacrylate resins. It determines their mechanical and physicochemical properties, and its reduction weakens the mechanical properties of a composite [41]. Shvero et al., by modifying the Filtek Supreme XT composite (3M ESPE Dental) with 2 wt.% QA-PEI-NPs did not observe a significant change in the DC, which increased from 51 to 53% after adding QA-PEI-NPs [35].

Beyth et al. studied the effect of incorporating 1 wt.% QA-PEI-NPs on the flexural properties of two commercial composites: Z250 (3M ESPE Dental) and Filtek Flow (3M ESPE Dental) [28]. The addition of QA-PEI-NPs to both materials resulted in a statistically insignificant reduction in the modulus. The flexural strength of the Z250 composite increased insignificantly, whereas that of the Filtek Flow composite decreased by approximately 40%.

**Scheme 1:**
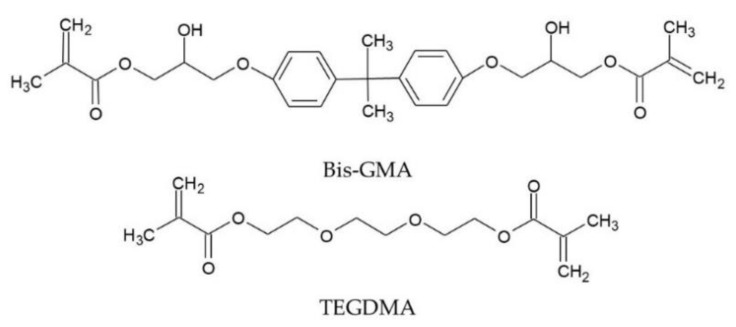
The chemical structures of Bis-GMA and TEGDMA.

This article aims to provide the lacking knowledge about the structure-property relationships of dental dimethacrylate polymer networks modified with QA-PEI-NPs. The research was carried out on the composition of two commercial dimethacrylate dental resins—60 wt.% bisphenol A glycerolate dimethacrylate (Bis-GMA) (Scheme 1) and 40 wt.% triethylene glycol dimethacrylate (TEGDMA) (Scheme 1) enriched with 0.5, 1, and 2 wt.% QA-PEI-NPs. The cured materials were tested for their degree of conversion (DC), density (*d_p_*), and polymerization shrinkage (S), glass transition temperature (T_g_), water sorption (WS), and solubility (SL), water contact angle (WCA), flexural modulus (E), flexural strength (σ), hardness (HB), and impact resistance (a_n_).

## 2. Materials and Methods

### 2.1. Materials

PEtOx (poly(2-ethyl-2-oxazoline), 50 kDa, Sigma Aldrich, St. Louis, MO, USA), HCl_aq_ (hydrochloric acid 37 wt.% in H_2_O, Acros Organics, Geel, Belgium), NaOH (sodium hydroxide, Chempur, Piekary Śl., Poland), diethyl ether (Chempur, Piekary Śl., Poland), 1,5-dibromopentane (Acros Organics, Geel, Belgium), 1-bromooctane (Acros Organics, Geel, Belgium), methyliodide (Acros Organics, Geel, Belgium), NaHCO_3_ (sodium bicarbonate, Chempur, Piekary Śl., Poland), anhydrous ethanol (Stanlab, Lublin, Poland), hexane (Chempur, Piekary Śl., Poland), Bis-GMA (bisphenol A glycerolate dimethacrylate, Sigma-Aldrich, St. Louis, MO, USA), TEGDMA (triethylene glycol dimethacrylate, Sigma-Aldrich, St. Louis, MO, USA), CQ (camphorquinone, Sigma-Aldrich, St. Louis, MO, USA), DMAEMA (dimethylaminoethyl methacrylate, Sigma-Aldrich, St. Louis, MO, USA) were used as received.

### 2.2. Synthesis

#### 2.2.1. Synthesis of Linear Polyethylenimine (PEI) 

**Scheme 2:**
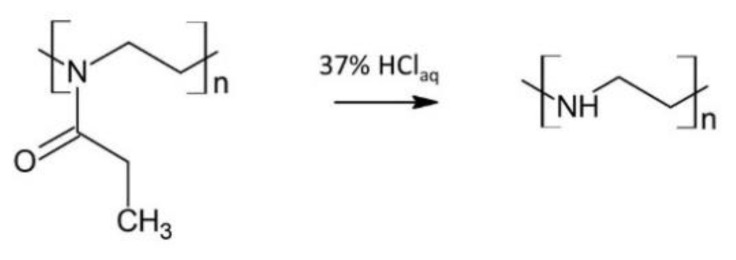
The PEI formation via the acidic hydrolysis of PEtOx.

Linear polyethylenimine (PEI) was obtained via the acidic hydrolysis of poly(2-ethyl-2-oxazoline) (PEtOx) (Scheme 2) [42].

PEtOx (5.03 g) was dissolved in 20 mL distilled water and added to a round-bottom flask equipped with a condenser, thermometer, and magnetic stirrer. Then, 15 mL of HCl_aq_ (37 wt.% in H_2_O) was added to the flask, and the reaction mixture was refluxed for 6 h with the use of the oil bath. After cooling to room temperature, unreacted HCl and newly-formed propionic acid were removed from the reaction mixture under reduced pressure. The powder residue was dissolved in distilled water, and the surplus acid was neutralized with 5 N NaOH solution to pH 9–10. The resulting white PEI precipitate was then recrystallized from distilled water, dissolved in methanol, and purified by precipitating into ice-cooled diethyl ether. After filtrating, pure PEI was dried under reduced pressure for 5 days.

#### 2.2.2. Synthesis of QA-PEI-NPs

QA-PEI-NPs were synthesized via an N-alkylation method [30]. The solution of PEI (1.77 g, 0.04 mol) in 20 mL absolute ethanol was introduced into a round-bottom flask equipped with a condenser, thermometer, and magnetic stirrer. Then, 1,5-dibromopentane (0.04 mol/mol PEI unit) was added, and the reaction was refluxed for 24 h with the use of the oil bath. In the second stage, the N-alkylation reaction was carried out. 1-bromooctane (1 mol/mol PEI unit) was added into the same flask, and the mixture was refluxed for 24 h. The resulting hydrobromic acid was neutralized with NaHCO_3_ (1.25 mol/mol 1-bromooctane) by heating under reflux for 24 h. The quaternization reaction was carried out in the same flask. The reaction mixture was refluxed for 48 h after adding iodomethane (3 mol/mol PEI unit). The resulting hydroiodic acid was neutralized with NaHCO_3_ (1 mol/mol PEI unit), by heating under reflux for 24 h. The QA-PEI-NPs were first purified by precipitation in distilled water. The filtered residue was then washed several times with hexane and distilled water, centrifuged, and lyophilized to dryness.

### 2.3. Curing Procedure

The 60 wt.% Bis-GMA and 40 wt.% TEGDMA composition was prepared and 0.4 wt.% CQ was added as a photoinitiator. The composition was divided into four parts. Three were admixed with 0.5, 1, and 2 wt.% QA-PEI-NPs. One composition was left unmodified for comparison purposes. Mixing was performed in a dark room, initially with a spatula, and then, resins enriched with QA-PEI-NPs were homogenized with an ultrasonic stirrer. A reducing agent—1 wt.% DMAEMA was admixed to each composition before its polymerization. All compositions were subjected to photocuring with a UV–VIS lamp, having 127 mm in diameter, and emitting UV–VIS radiation in the range from 280 to 780 nm with the exitance of 2400 mW/cm^2^ (Ultra Vitalux 300, Osram, Munich, Germany) for 1 h, from a distance of 15 cm. Polymerizations were carried out in square-shaped glass molds measuring 90 mm × 90 mm × 4 mm (length × width × thickness), Petri dishes measuring 40 mm × 4 mm (diameter × thickness), and disc-like Teflon molds measuring 15 mm × 1.5 mm (diameter × thickness). The oxygen inhibition was reduced by covering the polymerizing system with a PET film.

### 2.4. Instrumental Analysis

#### 2.4.1. Proton Nuclear Magnetic Resonance Spectroscopy (^1^H NMR) 

The 1H NMR spectra were collected with a 300 MHz NMR spectrometer (UNITY/INOVA, Varian, Palo Alto, CA, USA). PEtOx, PEI, and QA-PEI-NPs were analyzed in CDCl_3_, CD_3_OH, and DMSO solutions, respectively. TMS was used as an internal reference each time. 

#### 2.4.2. Infrared Spectroscopy (FTIR)

A Spectrum Two (Perkin-Elmer, Waltham, MA, USA) Fourier-transform infrared spectrometer (ATR-FTIR) was used in this study. The attenuated total reflectance sampling method was used to confirm the chemical structure of PEtOx, PEI, and QA-PEI-NPs. The same apparatus was used to characterize the monomer and polymer compositions enriched with QA-PEI-NPs. Uncured samples were analyzed as thin layers closed between two KBr pellets. The cured samples were powdered and sieved to a grain size less than 25 µm. They were analyzed as KBr pellets, one week after curing.

The FTIR spectra were used for the DC determination, using the following equation:

(1) $$DC\left(\%\right)=\left(1-\frac{{\left(\frac{A_{C=C}}{A_{Ar}}\right)}_{polymer}}{{\left(\frac{A_{C=C}}{A_{Ar}}\right)}_{monomer}}\right)\times 100$$

where A_C=C_ is the absorbance of the band at 1637 cm^−1^, corresponding to the C=C stretching vibrations in the methacrylate group, A_Ar_ is the absorbance of the band at 1608 cm^−1^, corresponding to the carbon-carbon stretching vibrations in the benzene ring.

#### 2.4.3. Dynamic Light Scattering (DLS) 

The QA-PEI-NPs sizes were measured at 25 °C by a dynamic light scattering instrument (DLS, Zetasizer Nano-S90, Malvern Technologies, Malvern, UK). Before measurements, QA-PEI-NPs were dispersed in distilled water and placed in a poly(methyl methacrylate) cuvette.

#### 2.4.4. Density and Polymerization Shrinkage

The liquid compositions were tested for density (*d_m_*) with a pyknometer according to ISO 1675 [43]. The cured materials were tested for density (*d_p_*) utilizing an analytical balance equipped with a density determination kit operating on Archimedes’ principle. The 0.01 mg analytical balance (XP Balance, Mettler Toledo, Greifensee, Switzerland) was used for measuring sample mass. 

The polymerization shrinkage (S) was calculated with the following equation:

(2) $$\;\;S\left(\%\right)=\left(1-\frac{d_{m}}{d_{p}}\right)\times 100$$

where *d_m_* is the monomer density, and *d_p_* is the polymer density.

#### 2.4.5. Differential Scanning Calorimetry (DSC)

The glass transition temperature (T_g_) was determined utilizing differential scanning calorimeter DSC 822^e^ (Mettler Toledo, Greifensee, Switzerland), following the procedure described in the standard ISO 11357-2:2020 [44]. Samples of cured materials weighed about 2.5 mg. Experiments were carried out in the air, at the temperature range from −40 to 200 °C, with a heating rate of 10 K/min. 

#### 2.4.6. Water Sorption and Solubility 

The water sorption (WS) and solubility (SL) were determined following the procedure described in the standard ISO 4049 [45], utilizing a 0.01 mg analytical balance (XP Balance, Mettler Toledo, Greifensee, Switzerland). Disc-like specimens of cured materials measuring 15 mm × 1.5 mm (diameter × thickness) were dried at 100 °C to constant mass (*m*_0_). The specimens were then introduced into glass containers with distilled water and kept for 7 days at room temperature. Just after removing from water, the specimens were thoroughly dried with absorbing paper and weighed (*m*_1_).

The WS was calculated with the following equation:

(3)   WS(μgmm3)=m1−m0V

where *m*_1_ is the mass of the swollen specimen, *m*_0_ is the initial mass of the dried specimen, and *V* is the initial volume of the dried specimen. 

The *SL* was determined by drying those specimens to a constant mass (*m*_2_), and calculating its value with the following equation:

(4)   SL(μgmm3)=m0−m2V

where *m*_2_ is the mass of the dried specimen after water storage.

#### 2.4.7. Water Contact Angle

The cured materials were tested for the water contact angle (WCA) utilizing a goniometer (OCA 15EC, Data Physics, Filderstadt, Germany). Deionized water (4 µL) was dropped onto the tested surface via the sessile drop method.

#### 2.4.8. Mechanical Properties

##### Flexural Properties

The flexural modulus (E) and flexural strength (σ) were tested using a universal testing machine Zwick Z020 (Ulm, Germany), following the procedure described in the standard ISO 178 [46]. Specimens of cured materials, measuring 80 mm × 10 mm × 4 mm (length × width × thickness) were cut out from the pre-formulated molds and sanded clean. E and σ were calculated with the following equations:

(5) $$\;\;\;E\;\left(MPa\right)=\frac{P_{1}l^{3}}{4bd^{3}\delta }$$

and

(6) $$\;\;\;\sigma \left(MPa\right)=\frac{3Pl}{2bd^{2}}$$

where *P*_1_ is the load at the selected point of the elastic region of the stress-strain plot, *P* is the maximum load, *l* is the distance between supports, *b* is the sample width, *d* is the sample thickness, and *δ* is the deflection of the sample at *P*_1_.

##### Hardness

The hardness (HB) was determined utilizing a VEB Werkstoffprűfmaschinen machine (Leipzig, Germany), following the procedure described in the standard ISO 2039-1 [47]. The disc-like specimens of cured materials, measuring 40 mm × 4 mm (diameter × thickness) were sanded clean before testing. 

HB was calculated with the equation:

(7) $$\;\;\;\;HB\left(MPa\right)=\frac{F_{m}\frac{0.21}{\left(h-h_{r}\right)+0.21}}{\pi dh_{r}}$$

where *F_m_* is the test load, *d* is the diameter of the ball intender (*d* = 5 mm), *h* is the immersion depth, and *h_r_* is the reduced depth of immersion (*h_r_* = 0.25 mm).

##### Impact Strength

The impact strength (*a_n_*) was determined utilizing the Charpy impact tester (Zwick RKP 450, Ulm, Germany), according to the standard ISO 179-1 [48]. Tests were performed on specimens of cured materials measuring 80 mm × 10 mm × 4 mm (length × width × thickness), which were cut out from the pre-formulated molds and sanded clean. 

*a_n_* was calculated with the equation:

(8) an(kJm2)=Anbh

where *A_n_* is the force causing fracture of a specimen, *b* is the specimen width, and *h* is the specimen thickness.

### 2.5. Statistical Analysis

A set of results achieved for five specimens was analyzed using the Statistica 13.1 software (TIBCO Software Inc., Palo Alto, CA, USA). The one-way ANOVA with Tukey’s HSD post hoc tests were performed (α = 0.05). The results were expressed as mean values, and their associated standard deviations (SD).

## 3. Results

**Figure 2:**
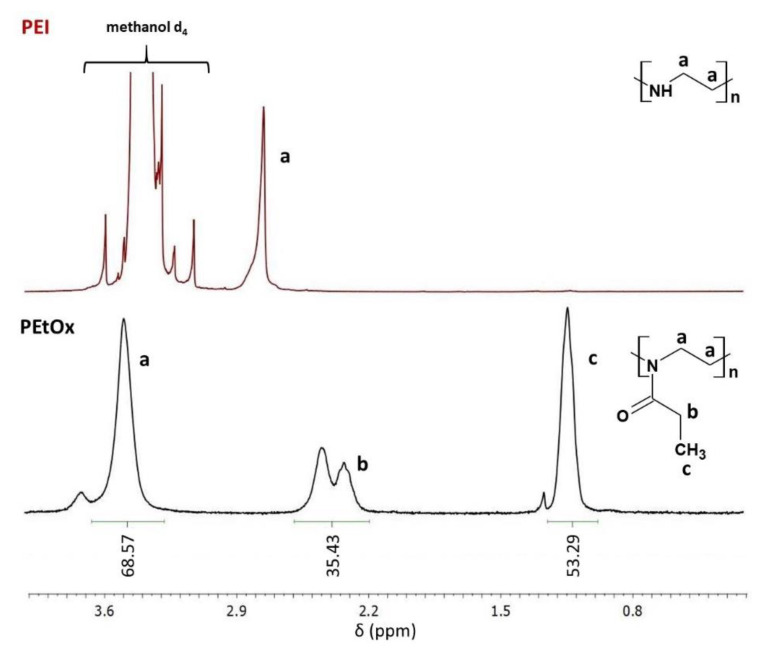
The ^1^H NMR spectra of PEtOx and PEI.

**Figure 3:**
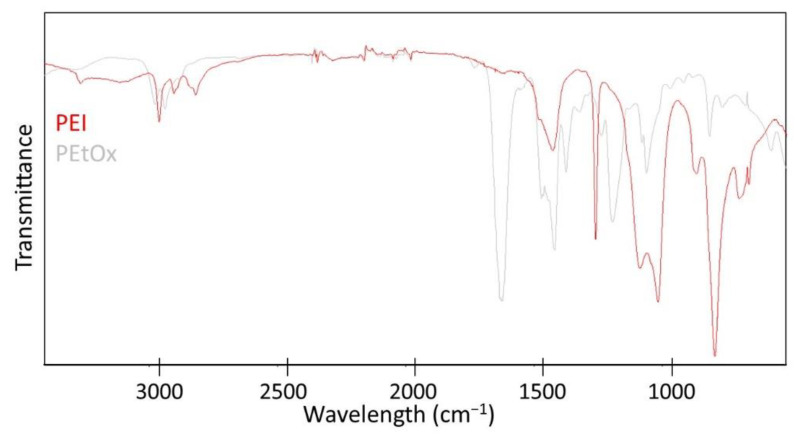
The ATR-FTIR spectra of PEtOx and PEI.

In this work, the composition of two common dental resins 60 wt.% Bis-GMA and 40 wt.% TEGDMA was modified by the physical admixing of QA-PEI-NPs. The latter was synthesized according to procedures described in the literature. First, linear PEI was obtained via the acidic hydrolysis of PEtOx [42]. The formation of PEI was confirmed by 1H NMR and ATR-FTIR measurements. The 1H NMR spectrum of PEI revealed the disappearance of signals from protons present in the -CH_2_CH_3_ PEtOx substituent, located at 1.13, 2.31, and 2.42 ppm (Figure 2). It also indicates that PEtOx hydrolysis was complete. Since the local chemical environment surrounding the hydrogen nuclei of –CH_2_CH_2_– groups was changed, they gave a single peak located at 2.73 ppm. The ATR-FTIR spectrum of PEtOx exhibited an absorption peak of the >C=O group in the amide group located at 1646 cm^−1^, which disappeared as PEI was formed. A peak of the –NH– group located at 3357 cm^−1^ was observed instead (Figure 3).

**Figure 4:**
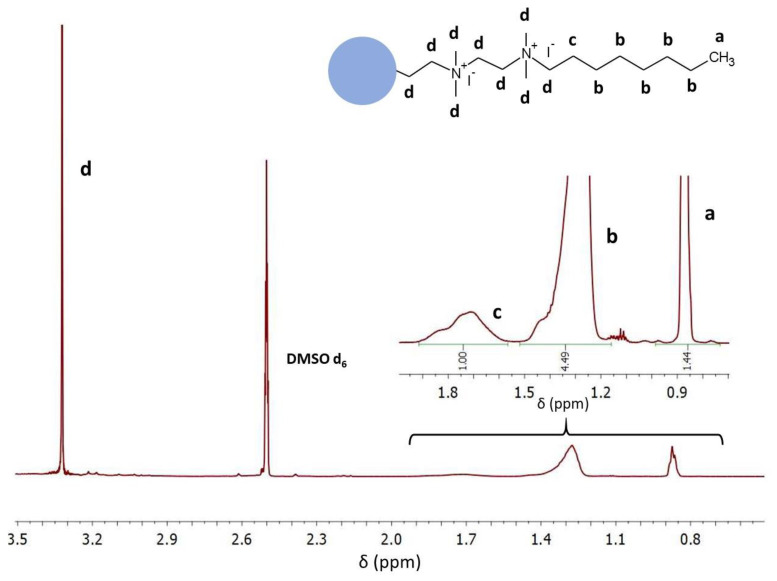
The ^1^H NMR spectrum of QA-PEI-NPs.

**Figure 5:**
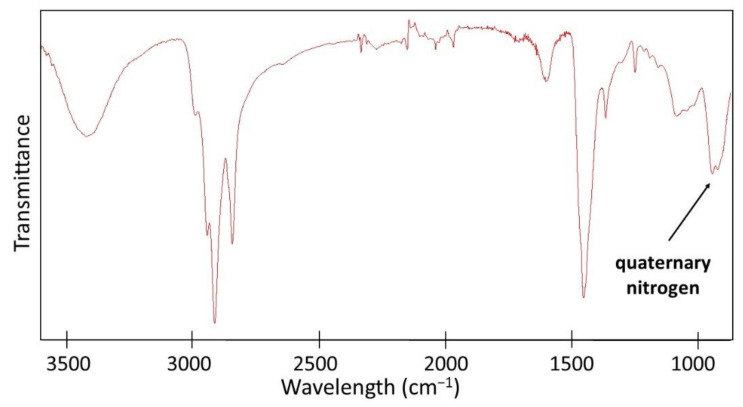
The ATR-FTIR spectra of QA-PEI-NPs.

In the second stage, PEI was crosslinked with 1,5-dibromopentane (0.04 mol/mol PEI unit), N-alkylated with 1-bromooctane (1 mol/mol PEI unit), and quaternized with iodomethane (3 mol/mol PEI unit). The formation of QA-PEI-NPs was confirmed by 1H NMR and ATR-FTIR measurements. The signals at 0.87, 1.20–1.50, and 1.72 ppm from protons of the bromooctane substituent (–CH_3_ and –(CH_2_)_6_– groups) were present in the 1H NMR spectrum of QA-PEI-NPs (Figure 4), but they were not observed in the 1H NMR spectrum of PEI (Figure 2). The signal coming from the -CH_2_- groups of PEI changed its position and moved from 2.74 ppm to 3.32 ppm due to changes in the chemical environment surrounding the hydrogen nuclei. It overlapped with the signals of the remaining –CH_2_– group of the octyl chain and -CH_3_ groups neighboring the quaternary nitrogen (Figure 4). The ATR-FTIR spectrum of QA-PEI-NPs exhibited characteristic absorption peaks of the amine group (3446 and 1620 cm^−1^), –CH_3_ and –CH_2_– groups (2954, 2925, 2852, and 1465 cm^−1^), and quaternary nitrogen (956 cm^−1^) (Figure 5).

**Figure 6:**
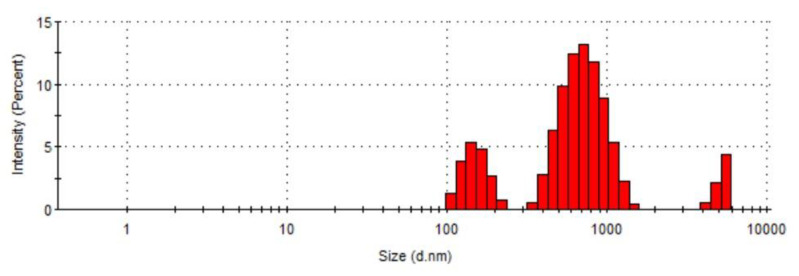
The DLS size distribution profile of QA-PEI-NPs.

The dimensions of the QA-PEI-NPs were analyzed utilizing the DLS technique (Figure 6). Three fractions of particles were detected with an average size of (i) 151 nm (±28 nm) that constituted 18.9% of all particles, (ii) 732 nm (±222 nm) that constituted 73.9% of all particles, and (iii) 5212 nm (±467 nm) that constituted 7.2% of all nanoparticles. The Z-average size was 509.1 nm.

The obtained QA-PEI-NPs were introduced into the Bis-GMA/TEGDMA composition in the amounts of 0.5, 1, and 2 wt.%. The modified compositions, as well as one free of QA-PEI-NPs, were hardened by photocuring with a common dental initiating system. Prepared materials were tested for selected physicochemical and mechanical properties.

In Table 1, the results of density and polymerization shrinkage are summarized. As can be seen, the density of uncured compositions (*d_m_*) did not change after adding QA-PEI-NPs (1.13 g/cm^3^). Curing increased the density (*d_p_*), which ranged from 1.22 to 1.24 g/cm^3^ and slightly increased upon increasing the concentration of QA-PEI-NPs; however, the neat polymer and the material containing 0.5 wt.% QA-PEI-NPs had the same density. The addition of 1 wt.% QA-PEI-NPs resulted in a statistically insignificant (*p* > 0.05) increase in the *d_p_*. The addition of 2 wt.% QA-PEI-NPs caused a 2% increase in the *d_p_* compared with the neat polymer. The latter difference was statistically significant (*p* ≤ 0.05).

The results in Table 1 also indicate that there is a relationship between the amount of QA-PEI-NPs and polymerization shrinkage (S). The S values ranged from 7.37 to 8.87% and increased as the concentration of QA-PEI-NPs increased. Compared with the neat polymer, this parameter first increased when the concentration of QA-PEI-NPs exceeded 0.5 wt.%. The addition of 1 and 2 wt.% of QA-PEI-NPs increased the S values, which were respectively, statistically insignificant (*p* > 0.05) and statistically significant (*p* ≤ 0.05). The latter increase was 20%.

**Figure 7:**
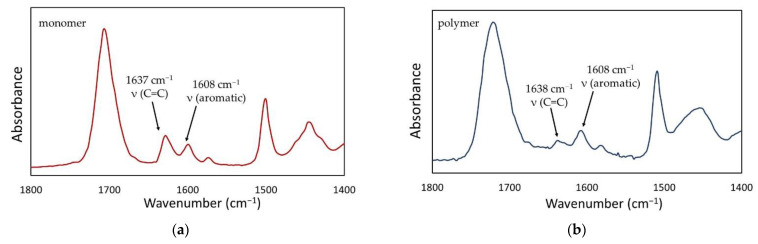
The representative FTIR spectra of the Bis-GMA/TEGDMA composition containing 0.5 wt.% QA-PEI-NPs in its uncured (**a**) and cured (**b**) forms.

**Figure 8:**
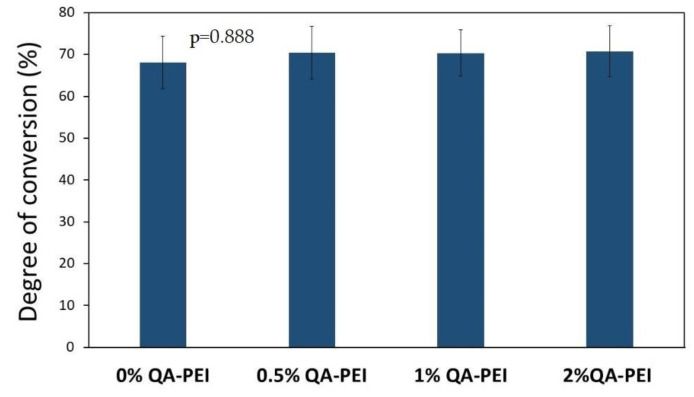
DC values of the materials. All the results for DC were statistically insignificant (*p* > 0.05).

The DC in cured materials was also determined using FTIR spectroscopy. Figure 7 shows the representative FTIR spectra of the composition containing 0.5 wt.% QA-PEI-NPs in its liquid and cured forms. The results for the DC are summarized in Figure 8. The neat polymer was characterized by a DC of 68.08%. Generally, the addition of QA-PEI-NPs increased the DC values, which ranged from 70.44 to 70.77%; however, all the differences in the DC values were not statistically significant (*p* > 0.05).

**Figure 9:**
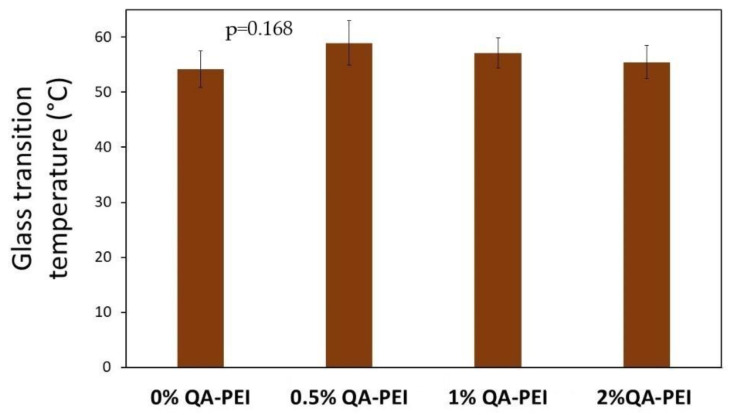
T_g_ values of the materials. All the results for T_g_ were statistically insignificant (*p* > 0.05).

In Figure 9, the T_g_ results are summarized. Generally, the T_g_ slightly increased due to the introduction of QA-PEI-NPs into the neat polymer (T_g_ = 54.19 °C) and ranged from 55.47 to 58.96 °C; however, the T_g_ decreased upon increasing the concentration of QA-PEI-NPs. All the differences in the T_g_ values did not have statistical meaning (*p* > 0.05).

**Figure 10:**
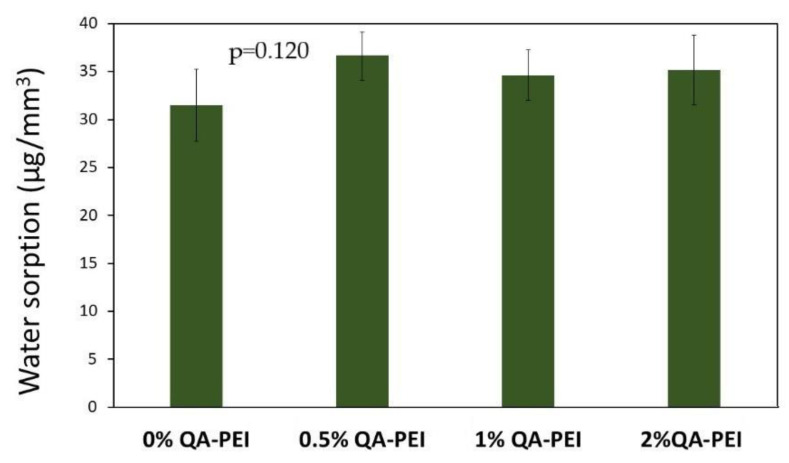
WS values of the materials. All the results for WS were statistically insignificant (*p* > 0.05).

In Figure 10 the results for water sorption (WS) are summarized. Its values slightly increased after the introduction of QA-PEI-NPs into the neat polymer (WS = 31.5 µg/mm^3^) and ranged from 34.63 to 36.59 µg/mm^3^; however, WS decreased upon increasing the concentration of QA-PEI-NPs. All the differences in the T_g_ values did not have statistical meaning (*p* > 0.05).

**Figure 11:**
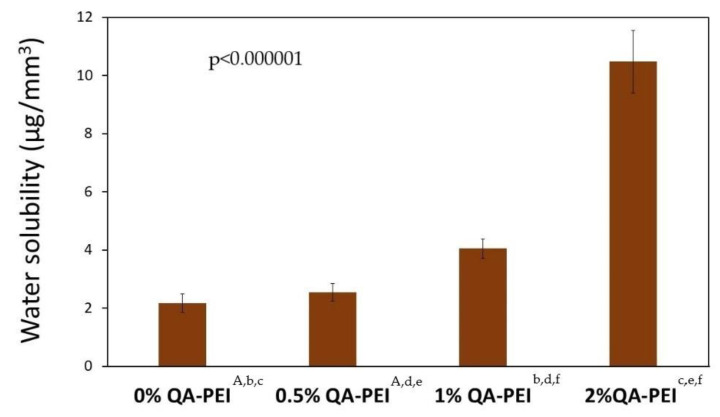
SL values of the materials. Statistically significant differences (*p* ≤ 0.05) are marked with the lower case letters, whereas statistically insignificant differences (*p* > 0.05) are marked with the upper case letters.

Figure 11 summarizes the water solubility (SL) results. Its values increased after QA-PEI-NPs were introduced into the neat polymer (SL = 2.17 µg/mm^3^) and ranged from 2.54 to 10.48 µg/mm^3^. In addition, SL increased upon increasing the concentration of QA-PEI-NPs. These increases were usually statistically significant (*p* ≤ 0.05). The only statistically insignificant difference (*p* > 0.05) in the SL values was found for the neat polymer and material containing 0.5 wt.% QA-PEI-NPs. This corresponded to 17% growth. The SL values of materials containing 1 and 2 wt.% QA-PEI-NPs were respectively 87% and 383% higher than the neat polymer.

**Figure 12:**
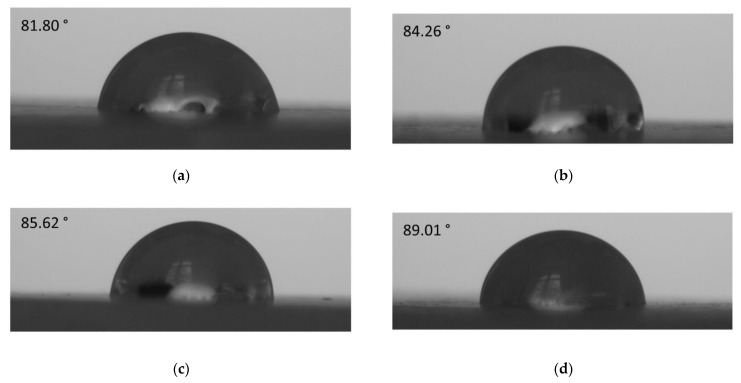
The images of deionized water droplets on the studied surfaces obtained from the goniometry camera for the (**a**) neat polymer, (**b**) material containing 0.5 wt.% QA-PEI-NPs, (**c**) material containing 1 wt.% QA-PEI-NPs, and (**d**) material containing 2 wt.% QA-PEI-NPs.

**Figure 13:**
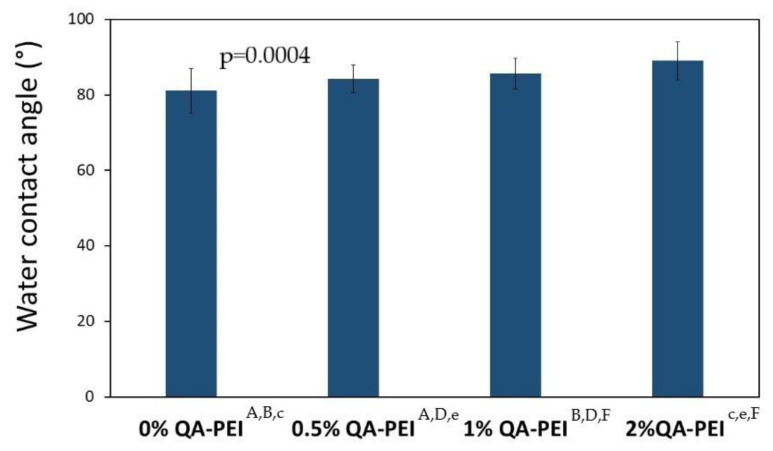
WCA values of the surfaces. Statistically significant differences (*p* ≤ 0.05) are marked with the lower case letters, whereas statistically insignificant differences (*p* > 0.05) are marked with the upper case letters.

The results for the water contact angle (WCA) are summarized in Figure 12 and Figure 13. The values increased due to the introduction of QA-PEI-NPs into the neat polymer (WCA = 81.08°) and ranged from 84.26 to 89.01°. They also increased upon increasing the concentration of QA-PEI-NPs. These increases usually did not show statistical significance (*p* > 0.05), except for the material containing 2 wt.% QA-PEI-NPs. The WCA of the latter material was statistically significantly higher (*p* ≤ 0.05), compared the neat polymer with as well as all the materials modified with 0.5 and 1 wt.% QA-PEI-NPs.

**Figure 14:**
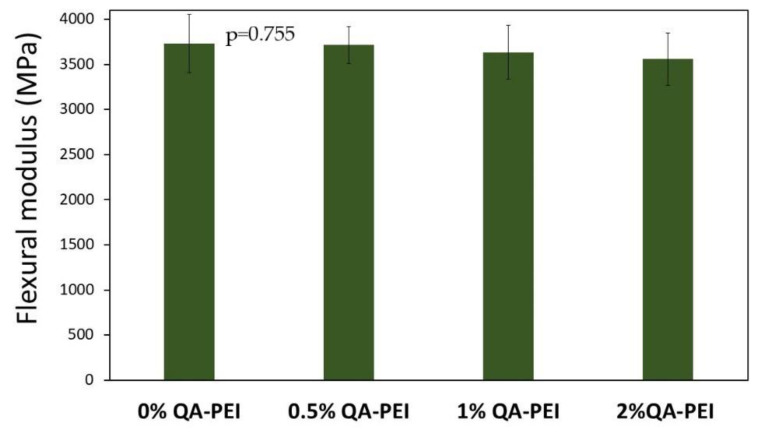
E values of the materials. All the results for E were statistically insignificant (*p* > 0.05).

In Figure 14, the results for the flexural modulus (E) are summarized. Its values decreased due to the introduction of QA-PEI-NPs into the neat polymer (E = 3731 MPa) and ranged from 3557 to 3712 MPa. They decreased upon increasing the QA-PEI-NPs concentration, but these changes were not statistically significant (*p* > 0.05). Compared with the neat polymer, the maximum reduction was only 5%, which was found for the material containing 2 wt.% QA-PEI-NPs.

**Figure 15:**
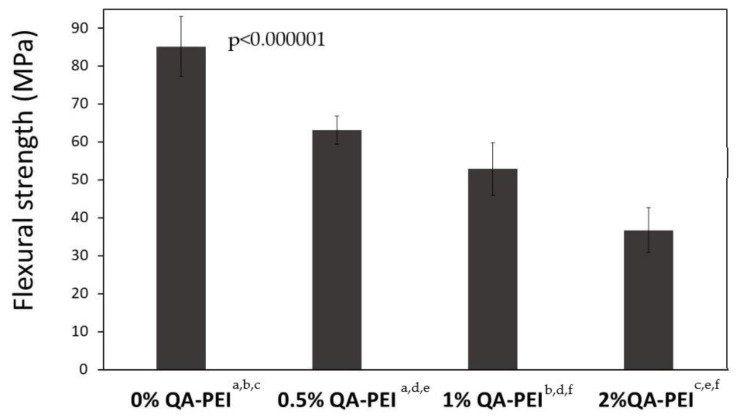
σ values of the materials. All the results for σ were statistically significant (*p* ≤ 0.05) and they are marked with the lower case letters.

In Figure 15, the results for the flexural strength (σ) are summarized. Its values decreased due to the introduction of QA-PEI-NPs into the neat polymer (σ = 85.18 MPa) and ranged from 36.76 to 63.09 MPa. They decreased upon increasing the concentration of QA-PEI-NPs, and all decreases were statistically significant (*p* ≤ 0.05). Compared with the neat polymer, the σ values of materials containing 0.5, 1, and 2 wt.% QA-PEI-NPs were 26, 38, and 57% lower, respectively.

**Figure 16:**
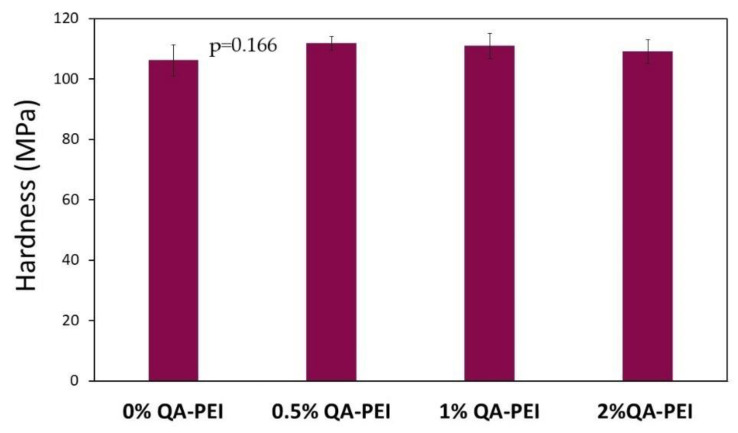
HB values of the materials. All the results for HB were statistically insignificant (*p* > 0.05).

In Figure 16, the hardness (HB) values are summarized, which slightly increased due to the introduction of QA-PEI-NPs into the neat polymer (HB = 106.19 MPa) and ranged from 109.1 to 111.81 MPa; however, the HB decreased upon increasing the concentration of QA-PEI-NPs. All the differences between HB values were statistically insignificant (*p* > 0.05).

**Figure 17:**
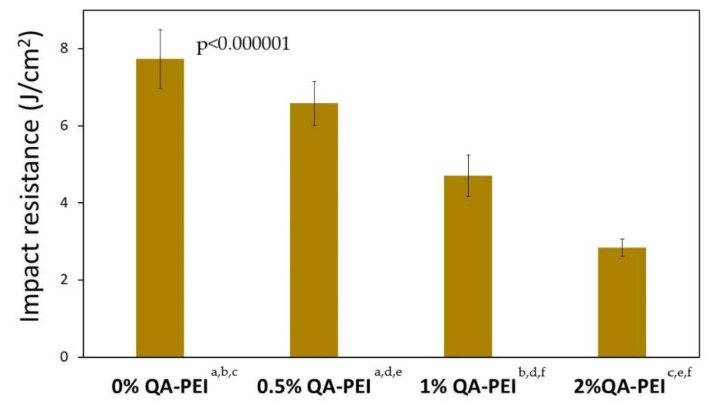
a_n_ values of the materials. All the results for a_n_ were statistically significant (*p* ≤ 0.05) and they are marked with the lower case letters.

In Figure 17, the impact strength (a_n_) values are summarized, which decreased due to the introduction of QA-PEI-NPs into the neat polymer (a_n_ = 7.73 J/cm^2^) and ranged from 2.84 to 6.58 J/cm^2^. They decreased upon increasing the concentration of QA-PEI-NPs, and all these decreases were statistically significant (*p* ≤ 0.05). Compared with the neat polymer, the a_n_ values of the materials containing 0.5, 1, and 2 wt.% QA-PEI-NPs were 15, 39, and 63% lower, respectively.

## 4. Discussion

The recent trends in dental restorative composites have shown huge interest in the development of materials possessing antibacterial activity. The physical admixing of bioactive compounds is one of the simplest ways to achieve this goal. As many of the most popular biocides show toxic effects, the application of QA-PEI-NPs seems to be a promising alternative. QA-PEI-NPs have already shown high antibacterial activity against many bacteria strains, including those responsible for tooth decay. When added to dental restorative composites, they successfully inhibit bacterial growth on their surfaces. Only 1 wt.% QA-PEI-NPs is sufficient to kill Gram-positive bacteria, whereas 2 wt.% is required to kill Gram-negative bacteria [1].

On the other hand, each physical modification of a material based on a polymer network can negatively affect its molecular structure, which can deteriorate the material’s physicochemical and mechanical properties; therefore, in this study, we investigated how the physical modification of the Bis-GMA/TEGDMA polymer network with QA-PEI-NPs affected its structural, physicochemical, and mechanical properties. The composition of Bis-GMA 60 wt.% and TEGDMA 40 wt.% was chosen as the most representative example of a dental composite restorative material matrix [41].

In this study, QA-PEI-NPs were synthesized starting from PEtOx hydrolysis, which resulted in the formation of linear PEI (Figure 2 and Figure 3), which was then subjected to crosslinking, N-alkylation, and quaternization. The latter reactions led to the formation of QA-PEI-NPs (Figure 4 and Figure 5). The DLS measurements revealed that achieved QA-PEI-NPs were 151 nm in average size. Bigger particles, with average diameters of 732 nm, and 5212 nm (Figure 6) can be recognized as agglomerates of QA-PEI-NPs [37]. This result is in agreement with the patent requirements, which states that QA-PEI-NPs should be from 10 to 10,000 nm in size. Preferred are particles less than 1000 nm in size, and most preferred are particles of up to 150 nm in size [27]. It is worth noting, that only 7.2% of all particle agglomerates were of about 5200 nm average size.

The photocured materials were tested for the degree of conversion. The FTIR analysis did not reveal any significant impact of the QA-PEI-NPs presence on the DC. If one looks at the DC results in more detail, a slight increase in its values can be observed. It might be attributed to the increase in loop and cycle formation [41]. However, it can be concluded that their influence on the DC can be neglected, when QA-PEI-NPs are added into the Bis-GMA/TEGDMA matrix at a maximum of 2 wt.%.

The DC is closely related to the polymerization shrinkage (S), which is an inherent effect of the polymerization process. During the curing reaction, the monomer molecules transform into repeating units, the van der Waals forces are converted into covalent bonds, and the polymerizing system undergoes volumetric contraction. As a result, the marginal gap formation takes place. It creates favorable conditions for biofilm development, resulting in the development of secondary caries and periodontal diseases [49]. In this study, the polymerization shrinkage was determined by measuring the density before and after curing. The density of uncured compositions (*d_m_*) did not alter with the addition of QA-PEI-NPs. In the case of cured materials, the addition of 0.5 wt.% QA-PEI-NPs also did not influence the density (*d_p_*). An increase in the concentration of QA-PEI-NPs resulted in a maximum 2% increase in this parameter (Table 1). As expected, the same relationship was observed for S. However, the scale of changes was greater. The neat polymer and material containing 0.5 wt.% QA-PEI-NPs were characterized by the same S value. An increase in the concentration of QA-PEI-NPs resulted in 10 and 20% increases in this parameter, respectively for samples containing 1 and 2 wt.% QA-PEI-NPs (Table 1).

The T_g_ of studied materials was determined as a crucial physicochemical factor of the polymer network constituting the dental composite matrix. A dental material must be able to withstand mechanical stress created by biting forces. It is only possible when the T_g_ exceeds the temperature in the mouth environment. The results showed that the modification of the Bis-GMA/TEGDMA composition with QA-PEI-NPs did not cause statistically meaningful changes in the T_g_ values. Since all tested materials had a T_g_ greater than 55 °C, it can be assumed that they would be in the glassy state once they were applied as a matrix in a dental composite.

Water sorption (WS) is another crucial physicochemical property of dental materials because it is responsible for their dimensional stability. Excess water swelling causes the dental material to expand, which, in extreme cases, can cause teeth fracture; therefore, the WS value of a dental material should be investigated, and its value should not exceed 40 µg/mm^3^ [45]. As can be seen from Figure 10, the WS values usually did not change significantly due to the addition of QA-PEI-NPs. The largest increase of 16% was observed with the addition of 0.5 wt.% QA-PEI-NPs; however, the highest WS value (36.59 µg/mm^3^) falls within the scope of the standard ISO 4049.

A dental material’s behavior in water is also usually characterized by its water solubility (SL). The maximum allowable soluble fraction is 7.5 μg/mm^3^ [45]. As can be seen from Figure 11, the SL value increased upon increasing the concentration of QA-PEI-NPs. The introduction of 0.5 wt.% QA-PEI-NPs negligibly increased the SL. Further increases in SL were significant and corresponded to 87 and 383% for materials containing, respectively 1 and 2 wt.% QA-PEI-NPs. The SL values of the materials containing 0.5 and 1 wt.% QA-PEI-NPs (2.54 and 4.05 µg/mm^3^, respectively) were lower than the limit defined in the standard ISO 4049. From this perspective, it can be concluded that the introduction of 0.5 and 1 wt.% QA-PEI-NPs into the Bis-GMA/TEGDMA matrix did not disqualify it from potential dental applications. The modification with 2 wt.% QA-PEI-NPs requires serious consideration because the SL value of this material was 40% higher than that specified in the standard ISO 4049. The results from the DC analysis suggest that such a significant increase in the SL values may be caused by the QA-PEI-NPs leaching. The general approach to the relationship between the DC and SL assumes that the lower the DC, the higher the SL [37]. As shown in Figure 8, the DC was not significantly influenced by the presence of QA-PEI-NPs; therefore, the evidence suggests that QA-PEI-NPs were not firmly anchored within the Bis-GMA/TEGDMA matrix. The interactions between QA-PEI-NPs and water molecules were probably stronger than those between QA-PEI-NPs and the Bis-GMA/TEGDMA polymer network. It also indicates that, in those interactions, the hydrophilicity of QA-PEI-NPs, resulting from the presence of the quaternary ammonium groups, dominated their hydrophobicity, resulting from the presence of the N-octyl substituents.

On the other hand, the water contact angle (WCA) results showed that the hydrophilicity of the tested surfaces decreased upon increasing the concentration of QA-PEI-NPs (Figure 13). These changes were usually insignificant, except for the material containing 2 wt.% QA-PEI-NPs, whose WCA increased by about 10% compared with that of the neat polymer; however, the WCA values of all tested surfaces were less than 90°, which classifies them as hydrophilic [50].

The Bis-GMA/TEGDMA materials enriched with QA-PEI-NPs were also tested for selected mechanical properties. They showed different responses to various mechanical factors.

The flexural modulus (E) of the studied materials changed insignificantly due to the addition of QA-PEI-NPs (Figure 14). Its values slightly decreased as the concentration of QA-PEI-NPs increased, but the maximum difference was only about 5% compared with the neat polymer. It might be attributed to the subtilities of the molecular structure of the Bis-GMA/TEGDMA polymer network. For example, an increase in the loop number could cause a decrease in E values (loops do not decrease the DC, but increase a network elasticity [41]).

Conversely, the flexural strength (σ) decreased significantly upon increasing the concentration of QA-PEI-NPs (Figure 15). Compared with the neat polymer, the σ decreased by 26, 37, and 57% for materials containing 0.5, 1, and 2 wt.% QA-PEI-NPs, respectively. A similar result was observed by Beyth et al. in a study on a commercial flowable composite composed of 53 wt.% Bis-GMA/TEGDMA matrix and 47 wt.% zirconia/silica filler [28]. They found that the addition of 1 wt.% QA-PEI-NPs decreased the flexural strength by about 40%. Interestingly, the flexural strength of another commercial material, composed of 40 wt.% Bis-GMA/UDMA/Bis-EMA matrix and 60 wt.% zirconia/silica filler, insignificantly increased. This leads to the conclusion that the influence of the modification of dental dimethacrylates with QA-PEI-NPs cannot be generalized to all kinds of materials, and it probably depends on their chemical composition. TEGDMA is a known factor causing mechanical strength deterioration. Due to the lack of hydrogen donors to hydrogen bonds its molecule cannot form strong physical crosslinks. On the contrary, UDMA can participate in strong hydrogen bonding and therefore increases mechanical strength. It explains why the Bis-GMA/UDMA/Bis-EMA/QA-PEI-NP composite was less sensitive to flexural stress than that comprising Bis-GMA/TEGDMA/QA-PEI-NP [28]. It also explains the result of our study, and allows us to assume that the introduction of QA-PEI-NPs into materials containing lower amounts of TEGDMA or those without TEGDMA will have a less significant impact on the flexural strength [41]. A certain improvement in flexural strength might be also achieved by the application of silanized fillers. They generally increase the flexural strength, in contrast to unsilanized fillers, which reduce it [51].

The hardness (HB) of the studied materials was not negatively affected by the addition of QA-PEI-NPs (Figure 16). The HB slightly increased (Figure 16). This result is in agreement with the literature data, which indicates that the hardness of dimethacrylate polymers is less sensitive to the DC and physical crosslinking [41].

Finally, the impact resistance (a_n_) of the studied materials was examined. This is a rarely investigated and problematic property because its values are usually lower than expected [52]. The a_n_ of studied materials decreased significantly upon increasing the concentration of QA-PEI-NPs (Figure 17). Compared with the neat polymer, the a_n_ value decreased by 15, 39, and 63% for materials containing 0.5, 1, and 2 wt.% QA-PEI-NPs, respectively. This behavior may be explained by the combination of several factors, including a low degree of physical crosslinking, incomplete conversion, formation of microgel agglomerates, weak intermolecular interactions between the Bis-GMA/TEGDMA matrix and QA-PEI-NPs [41,52,53], and even a presence of air bubbles trapped in a material [54]. Research is continuing in this area.

## 5. Conclusions

This article can be recognized as an initial study on the physicochemical and mechanical properties of dental polymers modified with QA-PEI-NPs. It provided a new portion of information about properties of the most basic dental composition, Bis-GMA/TEGDMA, modified with QA-PEI-NPs. The results of this study showed that the addition of QA-PEI-NPs into the Bis-GMA/TEGDMA composition up to a maximum of 2 wt.% did not negatively affect many of its properties, including the degree of conversion, polymerization shrinkage, glass transition temperature, water sorption, flexural modulus, and hardness. On the other hand, QA-PEI-NPs increased the water solubility, whereas the bending strength and impact resistance decreased. Therefore, it is worth extending the research on other dimethacrylate systems, comprising UDMA and/or Bis-EMA, as well as a filler, to achieve the optimum physicomechanical performance.

## Figures and Tables

**Table 1:** The monomer density (*d_m_*), polymer density (*d_p_*), and polymerization shrinkage (S) of studied materials. Statistically significant differences (*p* ≤ 0.05) are marked with the lower case letters, whereas statistically insignificant differences (*p* > 0.05) are marked with the upper case letters.

Sample Name	*d_m_* (g/cm^3^) (*p* = 0.9999)		*d_p_* (g/cm^3^) (*p* = 0.013)		S (%) (*p* = 0.007)	
	Average	SD	Average	SD	Average	SD
0% QA-PEI-NP	1.13	0.06	1.22 ^A,B,c^	0.01	7.37 ^A,B,c^	0.70
0.5% QA-PEI-NP	1.13	0.03	1.22 ^A,D,e^	0.01	7.37 ^A,D,e^	0.58
1% QA-PEI-NP	1.13	0.05	1.23 ^B,D,F^	0.01	8.13 ^B,D,F^	0.72
2% QA-PEI-NP	1.13	0.05	1.24 ^c,e,F^	0.01	8.87 ^c,e,F^	0.63

## Data Availability

Data supporting reported results are available from the authors.
